# Pathogenetic Gut Microbiota in Aortic Diseases: Evidence and Mechanisms Across Aneurysm, Dissection, and Inflammatory Aortopathies

**DOI:** 10.3390/nu18040565

**Published:** 2026-02-09

**Authors:** Leon Smółka, Miłosz Strugała, Karolina Blady, Bartosz Pomianowski, Karolina Kursa, Agata Stanek

**Affiliations:** 1 Student Scientific Association, Department of Internal, Metabolic Diseases and Angiology, Faculty of Health Sciences, Medical University of Silesia, Ziolowa 45/47 St., 40-635 Katowice, Poland; s83152@365.sum.edu.pl (M.S.); s86570@365.sum.edu.pl (K.B.); s83086@365.sum.edu.pl (B.P.); s91506@365.sum.edu.pl (K.K.); 2 Department of Internal, Metabolic Diseases and Angiology, Faculty of Health Sciences, Medical University of Silesia, Ziolowa 45/47 St., 40-635 Katowice, Poland; 3 Upper Silesian Medical Center, Medical University of Silesia, Ziolowa 45/47 St., 40-635 Katowice, Poland

**Keywords:** gut microbiota, intestinal barrier dysfunction, microbial translocation, abdominal aortic aneurysm, thoracic aortic aneurysm, aortic dissection, Takayasu arteritis, trimethylamine-N-oxide, short-chain fatty acids, lipopolysaccharide

## Abstract

Aortic diseases, including abdominal aortic aneurysm (AAA), thoracic aortic aneurysm (TAA), aortic dissection (AD), and Takayasu arteritis (TAK), are characterized by vascular remodeling and chronic immune–inflammatory activation, with AD often representing an acute complication of long-standing aortic wall vulnerability. Increasing evidence suggests that gut dysbiosis, impaired intestinal barrier integrity, and microbiota-derived metabolites may contribute to aortic wall injury. We synthesized current evidence linking the gut microbiome to aortic diseases and explored potential translational implications. PubMed, Scopus, and Web of Science were searched for microbiome-related studies on AAA, TAA, AD, and TAK published up to December 2025. Human observational and interventional studies were integrated with relevant experimental research. The strongest evidence was identified for AAA, where multiple cohorts report gut dysbiosis and reduced microbial diversity. Translational studies have detected bacterial DNA and microbial products in blood, aneurysm wall, or intraluminal thrombus, consistent with barrier-related microbial signaling and vascular inflammation, although these low-biomass findings do not establish microbial viability or causality. Microbiota-derived mediators—including trimethylamine-N-oxide, lipopolysaccharides, short-chain fatty acids, and bile acid derivatives—interact with pathways involved in cytokine signaling, oxidative stress, innate immune activation, and extracellular matrix degradation. Evidence for TAA and AD remains limited and suggests mainly modifier effects, whereas early studies in TAK indicate disease-associated microbiome and metabolite alterations. Mendelian randomization analyses have explored genetically proxied microbiome–AAA associations; however, results are heterogeneous, and causal inference remains provisional. Overall, the gut microbiome emerges as a plausible modifier of aortic disease, with the greatest translational relevance in AAA, highlighting the need for longitudinal multi-compartment studies and targeted interventions with aortic endpoints.

## 1. Introduction

Aortic diseases such as aneurysms and inflammatory aortopathies are chronic, progressive disorders characterized by long-standing vascular remodeling, extracellular matrix degradation, and persistent low-grade inflammation of the aortic wall, whereas aortic dissection is typically an acute event arising on the background of chronically vulnerable aortic tissue [1,2]. These conditions contribute substantially to cardiovascular morbidity and mortality through aneurysm enlargement, rupture, acute aortic syndromes, and irreversible end-organ damage, and they often coexist with chronic risk factors such as hypertension, diabetes, and atherosclerosis [1,3].

Over the last decade, the gut microbiota has emerged as a key modulator of cardiovascular homeostasis, influencing lipid metabolism, blood pressure regulation, endothelial function, and systemic inflammation through microbially derived metabolites and immune signaling pathways [4,5]. Dysbiosis has been consistently linked to hypertension, with experimental and clinical data showing that altered microbial composition and short-chain fatty acid (SCFA) signaling contribute to increased vascular tone, oxidative stress, and sympathetic activation [6,7,8,9]. In parallel, microbial metabolites such as trimethylamine-N-oxide (TMAO), bile acid derivatives, and tryptophan metabolites have been implicated in atherogenesis and vascular inflammation, suggesting a broad contribution of gut-derived signals to chronic arterial disease [5,10].

Evidence from atherosclerosis and peripheral artery disease further supports a gut–vascular axis, linking dysbiosis and gut-derived metabolites with arterial inflammation and clinical outcomes [11,12,13]. More recently, several clinical and translational studies have connected pathogenetic gut microbiota with aortic pathology, supporting the concept that the gut microbiome may influence aortic disease biology. In abdominal aortic aneurysm (AAA), sequencing studies and mechanistic work indicate gut dysbiosis and report detection of microbial DNA and/or microbial products beyond the gut (in blood and aneurysmal compartments) [14,15,16,17]. Parallel multi-omics studies in type A aortic dissection (AD) have reported distinct gut microbial signatures associated with inflammatory cytokines and metabolomic changes, while experimental models suggest that microbiota-derived metabolites such as indole-3-aldehyde and butyrate can attenuate dissection-related phenotypes via G protein-coupled receptors and anti-inflammatory signaling [18,19,20,21].

In inflammatory aortopathies, including Takayasu arteritis (TAK) and giant cell arteritis, blood- and vessel-associated microbiome profiles differ from healthy donors, and emerging data from vasculitis models suggest that intestinal dysbiosis may promote granulomatous vascular inflammation and aneurysm formation [22,23,24].

Together, these observations support the view that specific gut microbial communities and their metabolites may contribute to pathogenetic pathways shaping chronic aortic remodeling, inflammation, and thrombosis [3,14,20].

In this review, we synthesize current evidence on pathogenetic and potentially protective gut microbiota in aortic diseases, integrating human multi-omics data with experimental models. We focus on how specific taxa and microbial metabolites influence extracellular matrix degradation, vascular smooth muscle cell loss, immune activation, and thrombosis, and how these mechanisms interact with classical risk factors such as hypertension and genetic connective tissue disorders. This review outlines overarching mechanistic and taxonomic patterns, followed by disease-specific data for AAA, TAA, AD, and TAK, and concludes with translational perspectives and opportunities for microbiota-targeted prevention and therapy within chronic cardiovascular disease.

## 2. Materials and Methods

A structured literature search was conducted in PubMed, Scopus, and Web of Science from database inception to December 2025 to identify publications addressing associations between the gut microbiome and aortic diseases, including abdominal aortic aneurysm, thoracic aortic aneurysm, aortic dissection, and inflammatory aortopathies.

**Figure 1:**
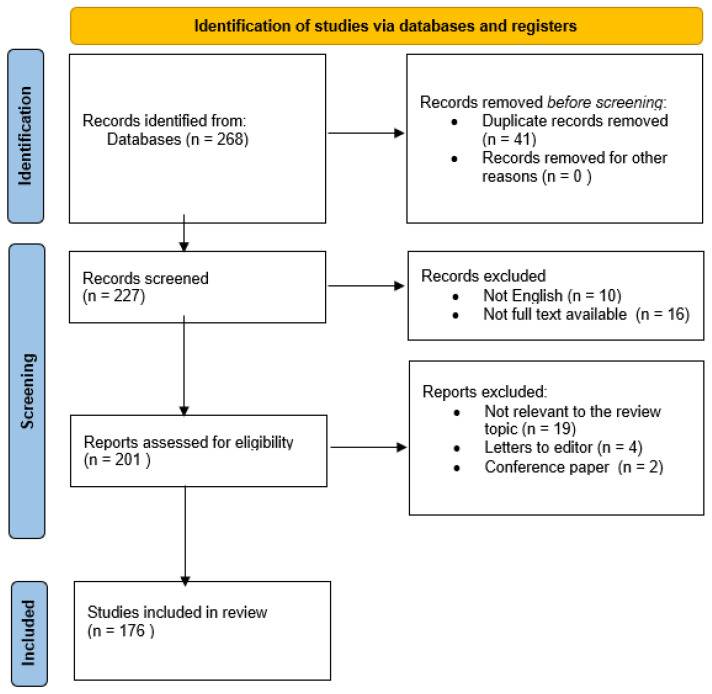
Flow diagram for study selection.

The search strategy combined terms related to the intestinal microbiome (e.g., gut microbiota/microbiome, dysbiosis, metagenomics, 16S rRNA) with terms related to aortic pathology and microbiota-linked mediators (e.g., TMAO, SCFAs, bile acids, LPS, gut barrier/permeability). Reference lists of selected eligible articles were additionally screened to identify potentially missed primary studies. Records retrieved from databases were deduplicated prior to screening. Titles and abstracts were screened first, followed by full-text assessment of potentially eligible reports. Non-English records and reports without full-text availability were excluded at the screening stage. During full-text assessment, reports were excluded if they were not relevant to the review topic or did not contain original data (e.g., letters to the editor, conference papers). The study selection process and reasons for exclusion are summarized in the flow diagram (Figure 1).

Eligible publications included original human studies (observational or interventional) and preclinical studies (animal and mechanistic experimental work) that assessed gut microbial composition and/or functional features (including metagenomic pathways) and/or microbiota-derived metabolites in relation to aortic disease outcomes (e.g., diagnosis, size/growth, dissection-related phenotypes, vascular inflammation, extracellular matrix remodeling, thrombosis). Extracted information included study design, population/model, microbiome assessment method, key taxa or functional pathways, metabolite-related findings, principal outcomes, and main study limitations. Evidence was synthesized narratively and organized into thematic domains covering microbiome alterations, microbial metabolites, barrier function, immuno-inflammatory mechanisms, and translational interventions.

## 3. Mechanistic Pathways: How Gut Bacteria Affect the Aortic Wall

### 3.1. Gut Dysbiosis, Intestinal Barrier Dysfunction, and Bacterial Translocation

Gut dysbiosis denotes qualitative and quantitative alterations in the intestinal microbial community, typically involving loss of beneficial commensals, reduced diversity, and expansion of pathobionts that promote chronic low-grade inflammation and metabolic stress [25,26,27]. Disruption of the intestinal barrier (“leaky gut”)—through changes in mucus, tight-junction integrity and local immune regulation—allows bacteria and their components, such as lipopolysaccharide (LPS), to translocate from the gut lumen into the portal and systemic circulation, driving abnormal immune activation in distant organs, including the vasculature [28,29,30]. Acute infectious illnesses with gastrointestinal involvement can transiently perturb gut microbiota composition and barrier/immune function, representing a potential short-term source of variability in microbiome studies [31,32,33]. Human data in aortic disease are consistent with this concept. Patients with aneurysmal aortic pathology show gut dysbiosis together with reported detection of bacterial DNA and/or microbial products in peripheral blood and aortic wall tissue, suggesting that microbial nucleic acids or inflammatory ligands may reach the vascular compartment [15,34]. Compartment-specific profiling of stool, blood, aneurysm wall, and intraluminal thrombus further supports partial overlap of microbial signals across sites, although these findings do not demonstrate microbial viability or prove translocation directionality [16,35]. Because these are low-biomass samples, detection of microbial DNA is vulnerable to contamination and does not establish viability, directionality, or causality.

Together, these data are consistent with a model in which dysbiosis and impaired barrier function are associated with the detection of bacterial products and nucleic acids in vascular compartments, which may engage pattern recognition receptors and sustain inflammatory signaling in aortic diseases; however, microbial viability, directionality of translocation, and causality remain to be established [15,16,25].

### 3.2. Microbial Metabolites and Vascular Processes

Gut microbes generate numerous bioactive metabolites and microbial products. Importantly, they produce trimethylamine (TMA) from dietary choline and L-carnitine, which is subsequently oxidized by host hepatic flavin-containing monooxygenases to TMAO, illustrating host–microbial co-metabolism [36,37]. In human studies and experimental models, higher plasma TMAO levels are associated with adverse vascular remodeling and enhanced aneurysm formation, with TMAO promoting vascular inflammation, oxidative stress, smooth muscle cell apoptosis, and ECM degradation [38,39,40,41].

**Figure 2:**
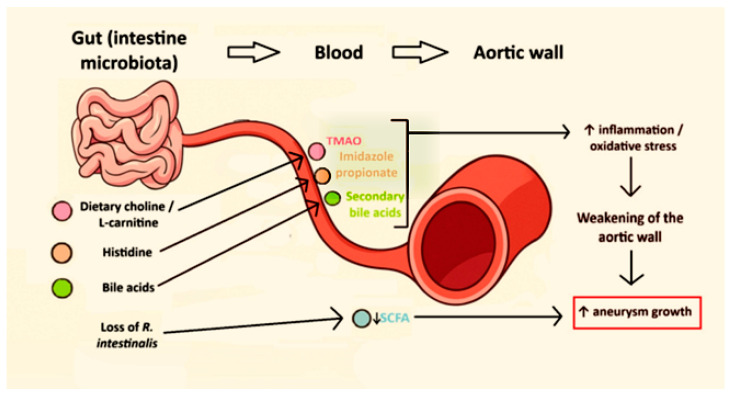
Schematic overview of microbiota-related mechanisms potentially involved in aortic aneurysm. Dietary choline/L-carnitine, histidine, and bile acids are metabolized by the intestinal microbiota into circulating precursors and metabolites (e.g., TMA, imidazole propionate, and secondary bile acids); TMA is subsequently oxidized by the host to trimethylamine-N-oxide (TMAO), which may influence inflammatory and oxidative pathways implicated in aortic wall remodeling. Reduced abundance of butyrate-producing taxa (e.g., *R. intestinalis*) and lower SCFA availability may further shift this balance toward a pro-aneurysmal milieu. Most mechanistic links are supported primarily by the experimental and broader cardiovascular literature; in human aortic disease, evidence is strongest for associative metabolite/taxa signals, rather than direct causal validation. Arrows indicate the proposed direction of metabolite translocation and associated downstream processes in the aortic wall. TMAO, trimethylamine-N-oxide; SCFA, short-chain fatty acid(s).

By contrast, SCFAs—particularly butyrate—are generally vasculoprotective. They are produced by fiber-fermenting commensals and signal via receptors such as GPR41 (G protein-coupled receptor 41) and GPR43 (G protein-coupled receptor 43) to modulate immune and vascular cell function, support barrier integrity, and dampen inflammation [42,43,44]. Loss of butyrate-producing bacteria, such as *Roseburia intestinalis*, and reduced butyrate levels have been linked to increased neutrophil infiltration, neutrophil extracellular trap (NET) formation, oxidative stress, and accelerated aneurysm growth in experimental models, whereas restoration of *R. intestinalis* or butyrate supplementation attenuates inflammation and limits lesion progression [45,46,47]. In parallel, gut-derived LPS acts as a potent innate immune stimulus that enhances monocyte and macrophage activation in vascular tissues, reinforcing inflammatory signaling pathways involved in aortic wall remodeling [36,48,49]. Microbial transformation of bile acids shapes a pool of secondary bile acids that signal through FXR (farnesoid X receptor) and TGR5 (Takeda G protein-coupled receptor 5), affecting lipid metabolism, oxidative stress, and inflammatory gene expression; disturbances in these pathways have been linked to atherosclerosis and may also influence aortic wall biology [50,51,52]. Imidazole propionate, a histidine-derived microbial metabolite previously associated with metabolic disease, has recently been identified as a driver of atherosclerosis and endothelial dysfunction, suggesting potential relevance for aortic disease [53,54,55]. Overall, a relatively small set of gut-derived metabolites—TMAO, SCFAs, bile acids, LPS, and imidazole propionate—appear capable of modulating inflammation, oxidative stress, thrombosis, blood pressure, and ECM turnover, all of which are central processes in aortic wall degeneration and predispose to aneurysm formation and dissection [36,38,45]. Figure 2 schematically summarizes the key gut-derived metabolites and their vascular effects, including pro-aneurysmal drivers and SCFA-related protective mechanisms.

### 3.3. Immuno-Inflammatory Pathways and Matrix Remodeling

Signals originating from gut dysbiosis and altered metabolite profiles converge on innate and adaptive immune pathways in the aortic wall. Gut-derived factors such as TMAO, LPS, and reduced SCFA availability can prime monocytes and neutrophils, skew macrophage polarization, and modulate T cell differentiation, thereby amplifying systemic and vascular inflammation [25,36,48]. Reviews integrating microbiota-derived metabolites further emphasize that TMAO, SCFAs, and bile acids shape monocyte, macrophage, and T cell phenotypes implicated in vascular injury [56,57,58].

In AAA, human tissue studies and experimental models consistently demonstrate dense infiltrates of macrophages, T cells, and neutrophils together with up-regulation of matrix metalloproteinases (MMP-2, MMP-9, and others), cathepsins, and pro-inflammatory cytokines, which drive elastin fragmentation, collagen remodeling, and smooth muscle cell apoptosis [59,60,61]. The neutrophil/NET axis appears to be a key interface between dysbiosis and this proteolytic remodeling. In a murine elastase model, and in patients with AAA, gut microbiome dysbiosis with loss of butyrate-producing bacteria promotes neutrophil recruitment, excessive NET formation, and up-regulation of inflammatory genes in the aortic wall, whereas restoration of *R. intestinalis* or butyrate supplementation attenuates these responses [45,62,63]. Basic and translational work on NET biology confirms that NETs amplify oxidative stress, endothelial damage, and matrix degradation in cardiovascular disease, including aortic aneurysm [64,65]. Several independent animal and human studies now link TMAO to this immune–proteolytic cascade in AAA. Experimental elevation of TMAO increases macrophage infiltration, expression of inflammatory and apoptotic genes, and MMP activity in aneurysmal aortas, resulting in more frequent and larger aneurysms, while pharmacological or dietary lowering of TMAO has the opposite effect [38,66,67].

**Figure 3:**
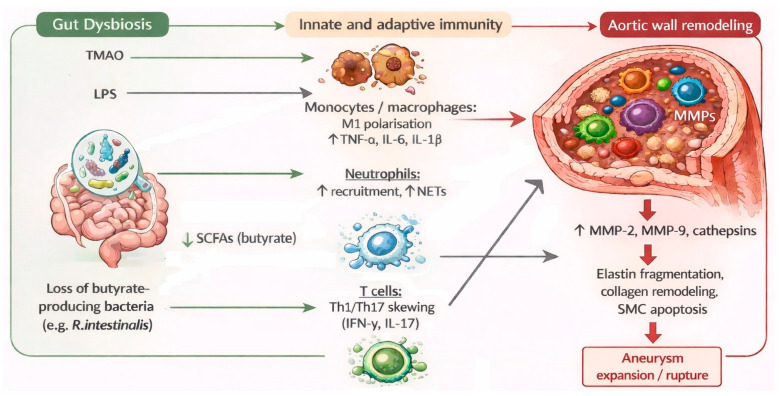
Proposed immuno-inflammatory links between gut dysbiosis and abdominal aortic aneurysm progression. Gut dysbiosis with increased TMAO and LPS and loss of butyrate-producing bacteria (*R. intestinalis*) leads to reduced SCFAs and activation of monocytes/macrophages (TNF-α, IL-6, IL-1β), neutrophils, and Th1/Th17 T cells (IFN-γ, IL-17). This pro-inflammatory and NET-rich milieu up-regulates matrix-degrading enzymes (MMP-2, MMP-9, cathepsins), causing elastin fragmentation, collagen remodeling, and smooth muscle cell apoptosis. The resulting weakening of the aortic wall promotes aortic aneurysm expansion and rupture. Elements such as the NET–butyrate–*R. intestinalis* axis are supported mainly by experimental models with limited direct validation in human aortic tissue. Arrows indicate the proposed direction of metabolite translocation and associated downstream processes in the aortic wall. TMAO—trimethylamine-N-oxide; LPS—lipopolysaccharide; SCFAs—short-chain fatty acids; *R. intestinalis*—*Roseburia intestinalis*; M1—classically activated (pro-inflammatory) macrophage phenotype; TNF-α—tumor necrosis factor alpha; IL-6—interleukin 6; IL-1β—interleukin 1 beta; NETs—neutrophil extracellular traps; Th1—T helper 1 cells; Th17—T helper 17 cells; IFN-γ—interferon gamma; IL-17—interleukin 17; MMPs—matrix metalloproteinases; MMP-2—matrix metalloproteinase 2; MMP-9—matrix metalloproteinase 9; SMC—smooth muscle cell.

Taken together, these data support an immuno-inflammatory model in which gut-derived signals reshape myeloid and lymphoid responses, enhance NET and MMP activities, and drive progressive degradation of elastin–collagen networks, thereby weakening the aortic wall and predisposing to aneurysm expansion and rupture [38,45,59]. The central components of this gut–immune–vascular cascade and their interactions are summarized schematically in Figure 3.

### 3.4. Blood Pressure, Hemodynamics, and the Microbiota

Hypertension is a major shared risk factor for AAA, TAA, and AD by increasing wall stress and accelerating medial degeneration. In a meta-analysis of cohort studies, hypertension increased the risk of developing AAA by approximately 66% [68,69,70]. A large-scale meta-analysis across Japanese and UK cohorts further demonstrated a dose-dependent association between blood pressure and both incidence and mortality of AD, with elevated risk even within the high–normal range [71,72,73]. These hemodynamic effects may interact with microbiota-driven mechanisms described above, linking dysbiosis to aortic disease progression through both biological and mechanical pathways.

There is increasing evidence that the gut microbiome contributes to blood pressure regulation. Experimental and clinical data summarized by Avery et al. show that changes in microbial composition, SCFA production, secondary bile acid signaling, and immune activation can raise or lower blood pressure [6,74,75]. A recent American Heart Association science advisory concluded that alterations in gut microbial composition and metabolite profiles are consistently linked to hypertension, and proposed microbiota-targeted strategies as a novel therapeutic approach [76,77,78].

SCFAs influence vascular tone, sympathetic nervous system activity, and renal sodium handling via receptors such as GPR41, GPR43, and Olfr78 (olfactory receptor 78), with measurable effects on blood pressure [79,80,81,82]. Hypertensive patients and experimental models often show reduced microbial diversity, altered SCFA profiles, and shifts in specific taxa, supporting a bidirectional link between dysbiosis and elevated blood pressure [83,84,85]. In the setting of aortic disease, this microbiota–blood pressure axis likely acts synergistically with the inflammatory and proteolytic mechanisms described above: dysbiosis not only promotes local immune activation and ECM degradation in the aortic wall, but also may contribute to systemic hypertension, thereby increasing mechanical load on a vulnerable vessel and potentially facilitating aneurysm enlargement and acute aortic events [6,68,71,86,87].

## 4. Gut Microbiota and Abdominal Aortic Aneurysm (AAA)

### 4.1. Clinical and Multi-Omics Evidence in Abdominal Aortic Aneurysm

AAA is a chronic, progressive vascular disease characterized by long-standing aortic wall remodeling, extracellular matrix degradation, and persistent low-grade inflammation, often remaining clinically silent until rupture or incidental detection [1,88,89]. In addition to established risk factors such as age, male sex, smoking, hypertension, and atherosclerosis, accumulating evidence suggests that alterations in the gut microbiota may act as a disease-modifying component in AAA pathogenesis [14]. Multiple clinical studies using stool-based 16S rRNA (16S ribosomal RNA) sequencing and metagenomic approaches have demonstrated significant differences in gut microbial composition between patients with AAA and healthy controls. These alterations include reduced microbial diversity, depletion of beneficial commensal bacteria, and enrichment of taxa associated with inflammation and metabolic dysregulation [90,91,92].

Importantly, evidence from human studies extends beyond fecal profiling and is consistent with microbial DNA/products being detectable beyond the gut in AAA. Bacterial DNA and microbial signatures have been detected in peripheral blood samples, as well as within aneurysmal wall tissue and intraluminal thrombus, suggesting that microbial nucleic acids and/or products may be detectable in vascular compartments [15,93,94]. Because blood, vessel wall tissue, and thrombus represent low-biomass compartments, these signals are particularly sensitive to contamination and batch effects. Detection of microbial DNA should therefore be interpreted as a marker of exposure/immune stimulation and does not establish viability, directional translocation, or causality. Detailed compartment-specific analyses comparing stool, blood, aneurysm wall, and thrombus microbiomes further support this concept. In patients with AAA, partial overlap has been observed between intestinal microbial profiles and those detected in vascular tissues and thrombus, suggesting selective enrichment and/or persistence of microbial DNA signals or components within the aneurysmal environment [16,95,96]. These vascular compartments are immunologically active and may amplify local inflammatory and proteolytic processes once exposed to microbial stimuli.

Recent work has expanded this paradigm by demonstrating that AAA is associated not only with bacterial dysbiosis, but also with alterations in the gut mycobiome. Multi-compartment profiling has revealed increased abundance of pro-inflammatory fungal taxa and reduced representation of potentially protective fungi in stool, blood, aneurysm wall, and thrombus samples from AAA patients [97,98,99]. Although derived from relatively small cohorts, these findings suggest that AAA reflects a broader disruption of the intestinal ecosystem, rather than isolated bacterial changes.

Another clinically relevant observation is the enrichment of oral-associated bacterial taxa in the gut microbiota of patients with AAA. Metagenomic analyses have identified increased abundance of species typically residing in the oral cavity within stool samples from AAA patients, suggesting an oralization-like shift in stool taxonomic profiles and/or enrichment of oral-associated taxa in the gut [91,100,101]. Given established links among oral dysbiosis, systemic inflammation, and cardiovascular disease, this oral–gut–aorta axis may represent an additional pathway contributing to chronic vascular inflammation in AAA [102,103,104]. However, alternative explanations include shared environmental exposures (diet, smoking), medication effects (e.g., antibiotics, PPIs, statins), and disease-related dietary changes that can reshape both oral and gut communities. Therefore, these compositional signals should be interpreted cautiously and do not, by themselves, establish directional oral-to-gut-to-aorta translocation.

Importantly, these observations are associative; microbial viability and causality remain to be established. Taken together, human clinical and multi-omics data indicate that AAA is associated with a distinct microbial signature characterized by gut dysbiosis and reported detection/compartmentalization of bacterial and fungal DNA signals or microbial products within the aneurysmal environment. These alterations align with inflammatory, proteolytic, and thrombotic processes implicated in aneurysm growth and rupture, supporting their potential biological relevance.

### 4.2. Experimental Abdominal Aortic Aneurysm Models and Microbiota Manipulation

Experimental models of AAA provide important translational support for clinical observations linking gut microbiota alterations with aneurysm development and progression. In widely used murine models, including angiotensin II (Ang II) infusion and elastase-induced aneurysm formation, induction of AAA is consistently accompanied by significant changes in gut microbial composition, mirroring key features observed in human disease [92].

In these models, AAA development is associated with reduced microbial diversity and enrichment of taxa linked to pro-inflammatory and proteolytic pathways, alongside impairment of intestinal barrier integrity. Such alterations are temporally related to aneurysm initiation and progression, suggesting that gut dysbiosis is not merely a secondary consequence of vascular injury, but may actively contribute to disease pathogenesis [46].

Importantly, direct manipulation of the gut microbiota modifies aneurysm outcomes in experimental settings. Depletion or enrichment of specific bacterial taxa alters aneurysm incidence, aortic diameter expansion, and rupture susceptibility. Restoration of beneficial commensal bacteria has been shown to attenuate inflammatory cell infiltration, reduce extracellular matrix degradation, and preserve vascular smooth muscle cell integrity within the aortic wall [45].

Several studies further demonstrate that modulation of microbial-derived oxidative and inflammatory signaling pathways influences AAA severity. Experimental interventions targeting microbiota-associated redox imbalance and inflammatory amplification reduce aneurysm progression, highlighting the functional relevance of gut-derived signals in vascular remodeling [105].

Dietary strategies that reshape gut microbiota composition also exert disease-modifying effects in AAA models. In particular, fiber- and inulin-enriched diets promote the expansion of beneficial bacterial populations, improve intestinal barrier function, and attenuate aneurysm growth, further supporting the concept that gut microbiota alterations can actively influence AAA pathobiology [106].

### 4.3. Mendelian Randomization and Causal Inference in Abdominal Aortic Aneurysm

While observational studies and experimental models strongly implicate gut microbiota alterations in AAA, Mendelian randomization (MR) analyses offer a complementary approach to infer causality by leveraging host genetic variants as instrumental variables. This approach can reduce confounding and reverse causation, but causal inference depends on instrument strength, taxonomic resolution, and pleiotropy; therefore, MR findings should be interpreted as hypothesis-generating. In addition, because microbiome composition is strongly shaped by environmental factors—particularly diet and medications—genetically proxied effects may be modest and context-dependent, contributing to heterogeneity across MR analyses [107]. Consistent with this caution, large-scale bidirectional MR analyses have reported that initial suggestive signals do not persist after FDR correction, underscoring the need for triangulation with longitudinal and mechanistic studies.

Recent MR studies have identified multiple gut microbial taxa that are genetically associated with AAA risk. These analyses suggest that increased abundance of certain bacterial genera is consistent with a causal contribution to higher susceptibility to aneurysm development, whereas other taxa may be consistent with protective effects. Importantly, several of these genetically inferred associations overlap with taxa identified in observational microbiome studies, reinforcing the biological plausibility of a contributory role for gut microbiota in AAA pathogenesis [107].

Beyond taxonomic signals, MR analyses integrating circulating metabolites provide further insight into potential mediators linking gut microbiota to AAA. Genetically predicted levels of specific microbial-derived metabolites have been associated with aneurysm risk, suggesting that metabolic pathways may represent key intermediates through which gut dysbiosis influences vascular remodeling and inflammation [108].

Additional MR studies examining broader cerebrovascular and cardiovascular outcomes support the concept that gut microbiota composition exerts systemic vascular effects, lending indirect support to its role in aneurysmal disease [109]. Although MR approaches are limited by available genetic instruments and population specificity, their convergence with clinical and experimental data supports continued investigation, while causality remains provisional given instrument limitations and potential pleiotropy.

### 4.4. Disease-Modifying Implications of Gut Microbiota in Abdominal Aortic Aneurysm

The convergence of clinical multi-omics data, experimental manipulation and MR supports a model in which gut microbiota dysbiosis acts as a disease-modifying factor in AAA, rather than a passive bystander. Across human studies, microbial alterations are consistently associated with inflammatory activation, extracellular matrix degradation, and thrombus formation, while experimental and genetic approaches further suggest a contributory role in aneurysm initiation and progression [14,107].

From a clinical perspective, these findings raise the possibility that gut microbiota signatures may serve as biomarkers for AAA presence, progression, or rupture risk. Microbial profiles detected in stool, blood, or aneurysmal tissue could complement imaging-based surveillance by providing information on inflammatory activity and biological disease aggressiveness, particularly in patients with small aneurysms under conservative management [16,110,111].

In addition, experimental data indicate that modulation of the gut microbiota can influence aneurysm severity, suggesting potential therapeutic relevance. Dietary interventions, microbiota-targeted strategies, and modulation of microbial-derived metabolic pathways may represent adjunctive approaches to conventional cardiovascular risk factor control, although their clinical applicability in AAA remains to be established [106,112]. Mechanistic evidence further supports a protective role of *Akkermansia muciniphila* in AAA. In an Ang II-induced AAA mouse model, fecal *A. muciniphila* abundance was reduced, while supplementation with *A. muciniphila* delayed aneurysm progression. This effect depended on endothelial PAS domain-containing protein 1 (EPAS1), which promoted transcription of CITED2 (CBP/p300-interacting transactivator with ED-rich tail 2); CITED2, in turn, reduced vascular smooth muscle cell (VSMC) apoptosis and dampened macrophage inflammatory responses, collectively limiting AAA development [113]. Notably, observational associations are not uniformly protective across datasets: in an experimental AAA setting, AAA diameter correlated with genus-level abundance, including *Akkermansia* [92]. This discrepancy may reflect stage-dependent ecology, confounding, or genus-level signals that mask strain- and context-specific functions. Accordingly, *Akkermansia* should be interpreted as a promising mechanistic, candidate rather than a consistent human biomarker.

Importantly, MR studies provide genetically inferred evidence consistent with a potential causal contribution of specific taxa or pathways to AAA risk, although signals vary and require biological validation [107,108]. Nevertheless, population specificity, limited genetic instruments, and the complexity of host–microbiota interactions necessitate cautious interpretation. Key studies are summarized in Table 1; however, the evidence base is currently weighted toward AAA, with fewer studies available for TAA, AD, and inflammatory aortopathies.

## 5. Gut Microbiota and Thoracic Aortic Aneurysm (TAA)

### 5.1. Human and Experimental Evidence Linking Gut Microbiota to TAA

TAA differs fundamentally from AAA with respect to etiology, anatomical context, and disease biology, with a stronger contribution of genetic predisposition, connective tissue disorders, and congenital valve abnormalities. Consequently, the body of evidence linking gut microbiota to TAA is considerably more limited than that available for AAA, and direct human data remain scarce [1]. Accordingly, unless explicitly stated as TAA-specific human evidence, mechanistic pathways discussed in this section should be interpreted as largely inferred from the AAA and broader cardiovascular literature. Bidirectional two-sample Mendelian randomization analyses exploring gut microbiome taxa and aortic aneurysm phenotypes report heterogeneous and generally modest genetically inferred signals consistent with potential causal effects for TAA compared with AAA, supporting the interpretation that current TAA-specific evidence remains limited and less consistent [114,115].

Available clinical studies suggest that patients with thoracic aortic pathology may exhibit gut microbiota alterations partially overlapping with those reported in AAA, including reduced microbial diversity and enrichment of pro-inflammatory taxa; however, these observations are largely extrapolated from mixed aneurysm cohorts or derived from indirect associations, rather than TAA-specific analyses [14]. In contrast to AAA, direct evidence of microbial translocation into the thoracic aortic wall or associated thrombus has not been robustly demonstrated in human TAA cohorts.

Experimental data addressing the role of gut microbiota in TAA are similarly limited. While animal models predisposed to thoracic aortic dilation demonstrate that systemic inflammation and metabolic perturbations can influence thoracic aortic remodeling, microbiota-specific interventions have rarely been evaluated in a TAA-focused manner. As a result, current mechanistic insights are primarily inferred from AAA models and broader vascular studies, rather than disease-specific experimentation [46,92].

### 5.2. Shared and Distinct Pathways Compared with AAA

Despite shared downstream features, such as vascular inflammation and extracellular matrix remodeling, AAA and TAA differ substantially in their upstream drivers. In AAA, gut microbiota dysbiosis, together with reported detection of microbial DNA/products beyond the gut, is linked to local inflammatory amplification, thrombus biology, and aneurysm progression in clinical and experimental studies, with complementary genetic evidence from MR analyses [16,107].

In contrast, TAA is more strongly influenced by intrinsic aortic wall defects, altered mechanotransduction, and genetic susceptibility, which may attenuate the relative contribution of gut-derived inflammatory signals. Nevertheless, systemic effects of gut microbiota—such as modulation of blood pressure, immune tone, and circulating metabolites—remain biologically relevant to thoracic aortic disease and may act as secondary disease modifiers [109].

### 5.3. Disease-Modifying Implications in TAA

Taken together, current evidence supports a model in which gut microbiota alterations act predominantly as modulatory, rather than primary, pathogenic factors in TAA. From a translational perspective, this suggests that microbiota-based interventions may have limited standalone efficacy in TAA, but could contribute to risk modification in selected patient subgroups, particularly those without known monogenic aortopathies [14].

## 6. Gut Microbiota and Aortic Dissection (AD)

### 6.1. Epidemiological and Clinical Associations

AD is an acute, life-threatening manifestation of aortic disease resulting from the interplay between pre-existing aortic wall vulnerability and sudden hemodynamic stress. Major risk factors include chronic hypertension, smoking, male sex, and systemic inflammatory states [1]. Importantly, many of these risk factors are increasingly recognized as being influenced by gut microbiota composition and microbial-derived metabolites.

Although direct microbiome profiling data in patients with AD are scarce, epidemiological and translational evidence supports an indirect association mediated through microbiota-related cardiovascular risk factors. Gut dysbiosis has been linked to blood pressure regulation, vascular stiffness, and systemic inflammatory tone, all of which critically modulate dissection risk [109]. In this context, microbial-derived metabolites affecting endothelial function and inflammation may contribute to increased susceptibility to acute aortic injury.

### 6.2. Translational and Experimental Insights

Experimental studies provide indirect mechanistic support for a contributory role of gut microbiota-associated pathways in vascular vulnerability relevant to AD. Gut dysbiosis has been shown to amplify systemic inflammation, oxidative stress, and endothelial dysfunction—processes that may weaken aortic wall integrity and lower the threshold for intimal disruption under acute hemodynamic stress [112]. In an Ang II-infused ApoE−/− mouse model, ursodeoxycholic acid (UDCA) attenuated acute AD formation and was associated with reduced oxidative stress and increased Nrf2 (nuclear factor erythroid 2-related factor 2) signaling, suggesting a bile acid/redox pathway that intersects with microbiota-related metabolism and may modify dissection-prone vascular stress in AD models [116].

Given the scarcity of direct human microbiome profiling in AD, the mechanistic links outlined below should be considered hypothesis-generating and are often extrapolated from aneurysm models and broader vascular biology. Although these observations derive primarily from aneurysmal disease models, rather than dissection-specific experiments, the implicated pathways substantially overlap with mechanisms known to predispose to AD. In addition, MR analyses linking genetically predicted gut microbiota composition and microbial-derived metabolites to vascular outcomes are consistent with systemic associations and a potential modifying role of gut microbiota-related signals in acute aortic pathology [108,109].

### 6.3. Knowledge Gaps and Translational Implications

At present, gut microbiota should be regarded as a potential modifier of AD risk, rather than an independent pathogenic driver. Importantly, a recent systematic review by Neiroukh et al. synthesized gut microbial taxa reported across abdominal aortic aneurysm, thoracic aortic aneurysm, and aortic dissection and identified substantial heterogeneity in study design, sampling compartments, sequencing platforms, and analytical pipelines [117]. The review highlighted limited overlap of reported taxa across studies and underscored that many associations lack replication across independent cohorts, reinforcing that currently reported microbiome signals in aortic diseases should be interpreted cautiously, primarily as associative and hypothesis-generating, rather than disease-specific causal signatures. The acute onset of dissection, limited availability of disease-specific tissue samples, and absence of longitudinal human microbiome studies represent major barriers to direct investigation [14,117].

## 7. Inflammatory Aortopathies (Takayasu Arteritis and Related Conditions)

### 7.1. Gut Microbiome and Multi-Omics Data in Takayasu Arteritis

TAK is a chronic large-vessel vasculitis affecting the aorta and its major branches, characterized by granulomatous inflammation and progressive arterial remodeling that can culminate in stenotic or aneurysmal complications. Current concepts frame TAK as a systemic immune-mediated disorder in which environmental and microbial exposures may interact with genetic susceptibility (HLA (human leukocyte antigen) and non-HLA loci) to sustain vascular inflammation over time [118,119,120].

Human multi-omics studies provide initial evidence that TAK is accompanied by gut microbial and metabolic shifts. An integrated 16S rRNA sequencing–metabolomics–lipidomics analysis reported reduced α-diversity, an altered Firmicutes/Bacteroidetes ratio, and enrichment of oral-derived or potentially pro-inflammatory taxa vs. controls, together with perturbations in amino acid, bile acid, and lipid pathways [121]. In a separate fecal microbiome study, dysbiosis with increased *Streptococcus* and *Campylobacter* was observed, and *Campylobacter gracilis* abundance independently associated with the presence and progression of aortic aneurysms, supporting the hypothesis that specific taxa may modify vascular risk within TAK [122].

Microbial signals linked to large-vessel vasculitis may extend beyond the gut. Peripheral blood microbiome profiling in TAK and giant cell arteritis (GCA) identified a distinct “vasculitis” signature enriched in selected Proteobacteria and Actinobacteria compared with healthy donors, suggesting partially shared microbial patterns across LVV (large-vessel vasculitis) phenotypes [22]. Because peripheral blood microbiome profiling is a low-biomass approach, these signals are particularly vulnerable to contamination and batch effects and should be interpreted as markers of exposure/immune stimulation, rather than evidence of viable organisms or directional translocation. Interpretation is strongest when rigorous negative controls, decontamination workflows, and replication across cohorts are reported. Reviews across connective tissue diseases and vasculitis consistently report depletion of SCFA-producing commensals and expansion of pathobionts in inflammatory vascular conditions, and experimental summaries propose that dysbiosis may amplify vascular inflammation and aneurysm formation—mechanistic themes that may be relevant to TAK [23,123,124].

Beyond microbiome profiling, metabolomics studies suggest that TAK is associated with measurable systemic metabolic reprogramming. NMR-based serum metabolomics discriminated TAK from healthy controls and revealed altered amino acid and energy metabolism metabolites that also correlated—at least in part—with disease activity [125]. Targeted follow-up work proposed that specific metabolite ratios (e.g., glutamine/glucose) may differentiate active from inactive disease and could serve as dynamic biomarkers reflecting immune–metabolic activity in TAK [126].

Consistent with this emerging multi-omics evidence base, biomarker-focused discussions in TAK increasingly integrate microbiota-related and metabolomic parameters alongside conventional inflammatory markers [127]. In pediatric TAK, a systematic overview similarly highlights biomarker candidates that reflect immune–metabolic crosstalk—compatible with a model in which dysbiosis-linked inflammation contributes to disease biology [128]. Proteomics and trans-omics studies further point to a shared LVV module involving innate immune activation, complement, and stromal/extracellular matrix remodeling—pathways that plausibly intersect with microbial products and host metabolic status [129,130].

Taken together, current human and synthesis-level evidence suggests that TAK is accompanied by gut dysbiosis—often described as enrichment of potentially pro-inflammatory taxa and depletion of beneficial commensals—together with systemic metabolomic and proteomic signatures consistent with immune activation and vascular remodeling [121,122,129,130]. Table 2 summarizes key primary studies reporting microbiome, metabolomic, and proteomic/trans-omics alterations in TAK and related large-vessel vasculitis.

### 7.2. Immunological Mechanisms and Aortic Remodeling in Inflammatory Aortopathies

Histopathology in TAK demonstrates granulomatous panarteritis with infiltrates of T cells, macrophages, and dendritic cells across the aortic wall, linking immune activation to intimal hyperplasia, medial injury, and adventitial fibrosis [118,120,131]. These immune programs provide the structural substrate for stenotic and aneurysmal phenotypes and frame where dysbiosis-associated mediators may act as disease modifiers, rather than primary drivers.

A consistent immunological feature of TAK is a Th1/Th17-skewed response (T helper 1/T helper 17). Increased circulating and tissue levels of IFN-γ, IL-6, and IL-17A have been reported, with cytokine profiles correlating with disease activity and vascular damage [132]. Th17.1/PD-1-related findings and broader cytokine signatures, including Th17/Treg imbalance and elevations in IL-6 and TNF-α, further support immunologically distinct inflammatory subsets in active disease [133,134,135,136].

Innate immune circuits also contribute substantially to vascular injury in TAK. Integrated bulk and single-cell transcriptomics show expanded pro-inflammatory monocytes/macrophages and activated cytotoxic lymphocytes, with gene programs enriched for antigen presentation, cytokine signaling, and matrix remodeling [137]. LVV frameworks further describe vasculitogenic CD4^+^ T cells and macrophage effector functions (including MMP production, oxidative stress, angiogenic signaling, and checkpoint dysregulation, such as reduced PD-1/PD-L1 signaling), while altered NK cell phenotypes have also been reported [138,139,140]. At the tissue level, chronic inflammatory injury in TAK is characterized by elastic lamina fragmentation, smooth muscle cell loss, and adventitial neovascularization—changes that create a permissive substrate for stenosis, dilatation, and aneurysm evolution [118]. Proteomic and trans-omics studies in LVV further link inflammation to ECM remodeling, including MMP-centered pathways (e.g., MMP-12), suggesting convergent remodeling programs across vasculitides [129,130].

Against this immunological background, intestinal dysbiosis has been proposed as a potential disease modifier of the Th1/Th17-dominant and macrophage-rich vascular inflammation observed in TAK. The human microbiome/omics observations in TAK, together with synthesis-level work revisiting microbial hypotheses in TAK and broader vasculitis-focused microbiome reviews, support a model in which loss of SCFA-producing commensals, expansion of pathobionts (including oral-derived taxa), and altered microbial metabolite profiles may shape dendritic cell activation and T cell polarization, while sustaining pro-inflammatory cytokine milieus relevant to arterial remodeling [23,121,122,123,141]. Inflammatory aortopathies such as TAK can be conceptualized as conditions in which dysbiosis-associated bacteria and microbiota-derived metabolites may interact with genetic predisposition and the local vascular immune niche, potentially reinforcing Th1/Th17-skewed and macrophage-dominated inflammation that may contribute to progressive aortic wall remodeling and divergent clinical phenotypes (stenotic and aneurysmal lesions).

**Figure 4:**
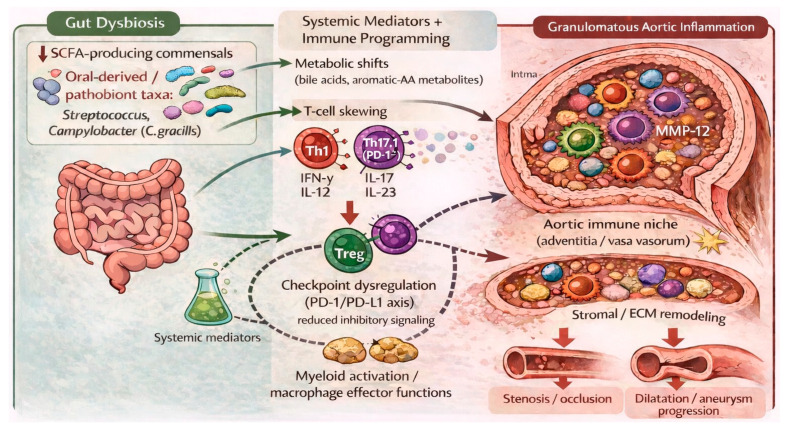
TAK/LVV-specific immune niche linking dysbiosis-driven systemic mediators and immune programming with granulomatous aortic inflammation and divergent remodeling phenotypes. Intestinal dysbiosis (reduced SCFA-producing commensals and enrichment of oral-derived/pathobiont taxa, including *Streptococcus* and *Campylobacter* (*C. gracilis*), is associated with systemic mediators and immune skewing (Th1/Th17/Th17.1), which converge in the adventitial/vasa vasorum immune niche to sustain granulomatous aortic inflammation. Downstream stromal/ECM remodeling (including MMP-12-linked signatures) promotes intimal hyperplasia with stenosis/occlusion or medial injury with dilatation/aneurysm progression. Solid arrows illustrate the proposed flow of events in the schematic, whereas dashed arrows indicate putative indirect or modulatory interactions.

The proposed immunological and structural processes linking intestinal dysbiosis with granulomatous inflammation and aortic remodeling in TAK are schematically summarized in Figure 4.

## 8. Diet and Microbiota-Targeted Interventions: Translational Perspectives in Aortic Disease

### 8.1. Dietary Patterns and Natural Bioactive Compounds

Dietary modification represents a feasible microbiota-modulating approach; however, in the context of AAA, direct human evidence linking specific dietary interventions to aortic endpoint outcomes remains limited. In the context of AAA, dietary patterns that support anti-inflammatory microbiome configurations—particularly those favoring SCFA production—may be relevant as an adjunct to overall cardiovascular risk reduction; however, effects on AAA incidence or progression in humans remain unproven [142,143]. Human evidence links long-term dietary patterns and anti-inflammatory dietary adherence to reproducible gut microbiome signatures and lower systemic inflammatory tone, supporting diet as a practical lever for systemic vascular inflammation; however, AAA-specific clinical endpoints have not been established [144,145,146].

A diet with microbiota-modulating potential should prioritize high dietary fiber intake. Reduced fiber consumption has been linked to a rising burden of inflammatory bowel diseases and autoimmune conditions in recent decades [142]. Increasing the intake of fiber-rich foods has been associated with greater gut microbiome diversity and a concomitant expansion of SCFA-producing bacteria, particularly butyrate producers [142,143,147]. Importantly, the potential relevance of fiber enrichment for AAA has been supported in animal models, where a high-fiber intervention was associated with attenuation of aneurysm progression alongside increased *Akkermansia* abundance and improved intestinal barrier function [106]. Fiber serves as a fermentable substrate for selected commensals (e.g., *Roseburia intestinalis*), thereby supporting SCFA biosynthesis [148,149,150]. Micronutrients may also shape host–microbe interactions; vitamin D status and supplementation have been associated with measurable microbiome changes alongside immune and barrier regulation [151,152,153]. A Mediterranean-style dietary pattern is frequently discussed as supportive of a health-associated gut microbiome, given its high contents of fiber, polyphenols, and unsaturated fatty acids. These components may shape the intestinal ecosystem through complementary mechanisms that favor anti-inflammatory microbial functions. Fiber provides a substrate for SCFA-producing taxa, whereas polyphenols can be metabolized into bioactive phenolic metabolites that may strengthen the intestinal barrier, exert anti-inflammatory effects, and increase SCFA production [146,154,155,156,157].

Polyunsaturated fatty acids, including ω-3: EPA (eicosapentaenoic acid), DHA (docosahexaenoic acid), and ALA (α-linolenic acid), have also been associated with microbiome modulation and can serve as substrates for specialized pro-resolving mediators such as resolvins, protectins, and maresins [158]. These mediators may promote resolution of inflammation via multiple mechanisms, including macrophage reprogramming (M1-to-M2), reduced neutrophil tissue recruitment, attenuation of pro-inflammatory cytokine production, and maintenance of intestinal barrier integrity [159]. In line with a microbiome–metabolite perspective, a clinical metagenomic analysis in patients with AAA reported reduced abundance of bacterial genes involved in α-lipoic acid biosynthesis (KEGG module IDs M00882–M00884, which are curated pathway definitions used for metagenomic functional annotation) [91]. While these findings are hypothesis-generating and do not establish causality, they support further investigation of diet-driven microbiome and metabolite modulation; however, translation to AAA prevention or progression in humans requires dedicated longitudinal studies and aortic endpoint trials. Beyond diet, structured exercise training improves endothelial function and exerts anti-inflammatory vascular effects, and should be considered alongside microbiota-targeted prevention frameworks [160,161,162].

### 8.2. Prebiotics, Probiotics, and Synbiotics

Modulation of the gut microbiota using prebiotics, probiotics, and synbiotics represents a non-invasive strategy with potential relevance for cardiometabolic risk factor modulation; however, robust AAA-specific endpoint trials are currently lacking. Current evidence does not allow any direct linkage between the use of prebiotics, probiotics, or synbiotics and attenuation of AAA progression. However, these interventions have been associated with favorable modulation of the gut microbiome and improvement of cardiovascular risk factors, including reductions in blood pressure [163], LDL (low-density lipoprotein) cholesterol and triglycerides [164], and fasting glucose [165]. These factors are also implicated in AAA development and progression; therefore, their improvement may offer indirect cardiometabolic benefit relevant to AAA risk profiles; however, aortic disease-specific outcomes remain insufficiently studied and should not be inferred from surrogate endpoints alone [166].

Prebiotics are non-digestible food components that are naturally present in foods or intentionally added, and—unlike probiotics—do not contain live microorganisms, but instead provide substrates that selectively support beneficial microbial growth and metabolism. A proof-of-concept preclinical intervention by Guo et al. demonstrated that dietary inulin supplementation increased *Akkermansia muciniphila* abundance, enhanced SCFA-related activity, and attenuated AAA progression in an animal model, alongside improvement of intestinal barrier function [106]. Although these data support mechanistic plausibility, human cohort studies and interventional trials are required to determine real-world efficacy and feasibility in AAA.

Probiotics are live bacterial cultures or yeasts administered to support restoration of a health-associated gut microbiome. Commonly used probiotic genera include *Lactobacillus* and *Bifidobacterium*, as well as the yeast *Saccharomyces boulardii*. Proposed mechanisms include competitive exclusion of potentially pathogenic taxa, reinforcement of intestinal barrier integrity, and promotion of anti-inflammatory microbial metabolites [167]. Direct evidence for probiotics in AAA remains limited; however, mechanistic considerations and broader AAA-focused microbiome syntheses outline plausible pathways through which probiotic-driven ecosystem shifts could intersect with aneurysm biology [14,45].

Synbiotics combine a probiotic with a prebiotic and are designed to improve survival and functional activity of administered microorganisms while simultaneously supporting beneficial resident taxa. Their anti-inflammatory effects have been reported across several autoimmune and inflammatory conditions [168,169]. Meta-analytic evidence indicates that synbiotic supplementation can reduce systemic inflammatory biomarkers, including CRP (C-reactive protein), TNF-α, and IL-6 [170]. Synbiotics have also been associated with improvements in cardiometabolic risk profiles in high-risk populations, which may indirectly intersect with AAA risk and progression, although aortic disease-specific endpoints remain largely untested [171].

Overall, the available data—while encouraging in terms of microbiome modulation and improvements in inflammatory and cardiometabolic markers—remain insufficient to draw firm conclusions regarding clinical benefits of prebiotics, probiotics, or synbiotics in AAA. Well-designed randomized trials with aortic endpoints are needed to clarify efficacy, target populations, and safety.

### 8.3. Postbiotics and Live Biotherapeutic Products

Postbiotics—preparations of non-viable microorganisms and/or their components—may represent an additional microbiota-targeted strategy with potential relevance to AAA risk modification and progression. Their lack of live bacteria may be advantageous in settings associated with impaired intestinal barrier integrity (e.g., inflammatory bowel disease) or immunosuppression, where theoretical concerns include translocation of viable organisms administered as probiotics or synbiotics [172]. Preclinical evidence supports the therapeutic plausibility of metabolite-centered approaches, including postbiotic formulations containing butyrate or propionate, where attenuation of AAA progression has been linked to expansion of Treg and reduction in NET formation, thereby potentially limiting protease-driven injury within the aortic wall [45,173].

Live biotherapeutic products (LBPs) are live organisms administered to humans to prevent, treat, or diagnose disease. In principle, LBPs could enable direct delivery of selected taxa with proposed beneficial effects in AAA-related contexts (e.g., *Akkermansia muciniphila*), thereby offering a targeted approach to reshape the intestinal ecosystem [174]. However, substantial translational challenges remain, including the need for appropriate delivery platforms and formulations, selection of strains and consortia, dosing, and quality control [175]. At present, evidence is insufficient to support routine use of LBPs in aortic disease, and further preclinical and human studies are required to define efficacy, safety, and clinically meaningful endpoints. In particular, human AAA endpoint trials are not available, and current support is primarily preclinical or extrapolated from broader cardiometabolic outcomes. Table 3 summarizes major microbiota-targeted product classes discussed in this section, highlighting their definitions, proposed mechanisms, evidence levels (with emphasis on AAA), and key translational limitations.

## 9. Strengths and Limitations

This review synthesizes clinical, experimental, and multi-omics evidence linking gut microbiota and microbiota-derived metabolites to major aortic disease phenotypes (AAA, TAA, AD, and inflammatory aortopathies). A key strength is the cross-disease framework, which highlights shared pathways (immune activation, extracellular matrix remodeling, and hemodynamic stress) while distinguishing phenotype-specific signals and translational implications. Limitations include the predominantly observational, cross-sectional nature of human studies, substantial heterogeneity in cohorts and analytical pipelines, and frequent confounding by diet, comorbidities, smoking, and medications (including antibiotics and immunomodulators). These sources of heterogeneity include differences in sequencing platforms, bioinformatic pipelines, sampling compartments, and covariate adjustment, which likely contribute to inconsistent taxon-level findings across cohorts and may also help explain variability in Mendelian randomization results. Many human microbiome studies are small and cross-sectional, limiting statistical power, increasing false-positive risk under multiple testing, and reducing reproducibility across cohorts. The microbiome literature is also susceptible to publication and positive result bias, and null findings may be underreported. This may inflate perceived effect sizes and contribute to limited reproducibility across cohorts. Similar gut microbiome–metabolite associations have also been reported in other vascular beds (e.g., atherosclerosis and peripheral artery disease), suggesting that part of the signal may reflect systemic vascular inflammation, rather than aorta-specific mechanisms. At the same time, aortic phenotypes (particularly AAA) have distinct wall biology and biomechanical stress, so vascular bed comparisons should be interpreted cautiously and validated with aortic endpoint-focused studies. An additional limitation is the challenge of low-biomass sequencing (e.g., blood/vessel tissue), where contamination and detection of microbial DNA without evidence of viability can complicate biological interpretation. Experimental models provide mechanistic insights, but may not fully translate to human pathology. Future work should prioritize harmonized multi-compartment sampling, longitudinal designs, and interventional studies with aortic endpoints to establish causality, clinical utility, and safety of microbiota-targeted approaches.

## 10. Conclusions

Current evidence links gut dysbiosis and microbiota-derived mediators to aortic disease biology via intestinal barrier perturbation, systemic inflammation, immune cell programming, and extracellular matrix remodeling. The evidence base is strongest for AAA, where human and experimental studies support microbiome–metabolite–immune interactions that may influence aneurysm progression. Data for TAA and AD are more limited and heterogeneous, suggesting microbiome-related signals may act mainly as disease modifiers, rather than primary drivers. In inflammatory aortopathies such as TAK, emerging microbiome and multi-omics findings are consistent with a model in which dysbiosis-associated systemic mediators may interact with a vascular immune niche and contribute to granulomatous inflammation and remodeling. Overall, microbiota signatures and microbial metabolites are promising candidates for biomarker development and adjunctive prevention/therapy, but clinically actionable translation requires longitudinal human studies and aortic endpoint-driven trials.

## Figures and Tables

**Table 1:** Study-level evidence linking gut microbiota taxa and microbial-derived metabolites with aortic diseases (AAA, TAA, AD). Evidence is currently most robust for AAA, with more limited and heterogeneous data for TAA and AD.

Study	Disease Context	Model/Population/Method	Main Microbiota/Metabolite Finding	Proposed Role/Translational Note
He et al. 2025 [112]	AAA	Animal + cell lines	*A. muciniphila* treatment prevented AAA development, reduced ECM degradation, and suppressed NET formation.	(Preclinical) *A. muciniphila* was associated with reduced NET-related injury and VSMC loss (via lower ferroptotic stress) in experimental AAA models; translation to human AAA remains unproven.
Wang et al. 2024 [113]	AAA	Animal model	Reduced fecal *A. muciniphila* in AAA; supplementation delayed AAA progression through an EPAS1 → CITED2 pathway.	(Preclinical/mechanistic) EPAS1/CITED2 axis activation was linked to reduced macrophage inflammatory signaling and VSMC apoptosis in experimental settings, suggesting a potential wall-protective mechanism that requires validation in human AAA.
Guo et al. 2025 [106]	AAA	Animal model	Inulin diet enriched *Akkermansia*, improved barrier markers, and reduced circulating LPS/IL-1β; gavage with *Akkermansia* also mitigated AAA.	(Preclinical) Barrier reinforcement was associated with lower endotoxemia and inflammatory recruitment, and reduced elastin injury, in AAA models; AAA-specific clinical endpoint data are lacking.
Tian et al. 2022 [45]	AAA	Human + animal model	AAA associated with reduced *Roseburia intestinalis*; *R. intestinalis* and butyrate reduced neutrophil infiltration/NETs and VSMC phenotypic switching.	(Preclinical) Butyrate/SCFA-related signaling (including *R. intestinalis* enrichment) was linked to reduced NET-mediated inflammation and VSMC dysfunction in models; direct validation in human aortic tissue is limited.
Ito et al. 2023 [90]	AAA	Human cohort	AAA patients had lower abundance of *Bifidobacterium adolescentis* (species-level) vs. controls.	(Observational, associative) Reduced abundance of putatively protective commensals may reflect a less anti-inflammatory microbiome state associated with AAA susceptibility; causality is not established.
Xie et al. 2020 [92]	AAA	Animal model	AAA mice showed gut dysbiosis; AAA diameter correlated with genera including *Akkermansia* (and others identified by LEfSe/correlation).	(Observational, associative; genus-level) Genus-level correlations with aneurysm diameter may reflect dysbiosis–severity coupling and/or confounding and do not establish protective vs. pathogenic directionality; stage effects should be considered.
Zhou et al. 2024 [17]	AAA	Human genetic MR	Bidirectional MR suggested taxa increasing AAA risk (e.g., *Bilophila*, *Catenibacterium*) and taxa decreasing risk (e.g., Lentisphaeria/Victivallales-related signals).	(MR; hypothesis-generating) Genetically proxied microbiota composition may relate to AAA risk via inflammatory/metabolite pathways; findings are heterogeneous and require hypothesis-driven validation and triangulation.
Tan et al. 2025 [109]	AAA/TAA/other aneurysms	Human genetic MR	Reported causal associations between specific taxa and aneurysm subtypes; for AAA, *Victivallaceae* protective, while *Bilophila*/*Catenibacterium* associated with higher risk.	(Synthesis/observational) Highlights candidate pro-inflammatory taxa as risk-associated signals and potentially protective taxa as preventive hypotheses; biological validation and replication across cohorts are needed.
Sun et al. 2024 [114]	AAA/TAA/AD	Human genetic MR	Large-scale bidirectional MR found no associations surviving FDR correction; initial “suggestive” signals did not remain significant after correction.	(MR; large-scale) No associations survived FDR correction in bidirectional MR; supports cautious interpretation of single-taxon causality and underscores the need for stronger instruments and mechanistic triangulation.
Qiu et al. 2024 [115]	AAA/TAA/IA/AA	Human genetic MR	Two-sample MR: Firmicutes/Lentisphaeria/Victivallales showed protective association with AAA risk; additional suggestive taxa across aneurysm types.	(Synthesis/observational) Suggests candidate protective multi-taxon patterns (e.g., Firmicutes-related signatures) as preventive hypotheses and emphasizes multi-taxon signatures over single organisms; requires replication and functional validation.
Liu et al. 2016 [116]	AD (experimental acute AD)	Animal model	UDCA reduced acute AD incidence and maximal aortic diameter, with reduced ROS and VSMC apoptosis via Nrf2-related antioxidant signaling.	(Mechanistic/extrapolated) Microbiota-adjacent bile acid signaling may modulate wall vulnerability (oxidative injury/VSMC stress) in dissection-relevant contexts; evidence in human AD remains limited and largely indirect.

AAA—abdominal aortic aneurysm; TAA—thoracic aortic aneurysm; AD—aortic dissection; MR—Mendelian randomization; NETs—neutrophil extracellular traps; VSMC—vascular smooth muscle cell; ECM—extracellular matrix; LPS—lipopolysaccharide; ROS—reactive oxygen species; UDCA—ursodeoxycholic acid; Nrf2—nuclear factor erythroid 2-related factor 2; FDR—false discovery rate.

**Table 2:** Study-level evidence linking microbiome and multi-omics signatures with TAK and related large-vessel vasculitis. Overall, available studies suggest reduced diversity and enrichment of potentially pro-inflammatory/oral-associated taxa alongside metabolomic/proteomic signatures consistent with immune activation and remodeling.

Study	Disease Context	Model/Population/Method	Main Microbiome/Omics Finding	Proposed Role/Translational Note
Fan et al. 2023 [121]	TAK vs. healthy controls	Stool 16S rRNA sequencing + serum metabolomics/lipidomics	Reduced α-diversity; altered Firmicutes/Bacteroidetes ratio; enrichment of oral-derived/potentially pro-inflammatory taxa; concurrent metabolomic and lipidomic pathway shifts	Multi-omics signature supports gut–systemic metabolic coupling; candidate biomarker framework
Manabe et al. 2023 [122]	TAK with vs. without aortic aneurysm; progression analysis	Stool microbiome profiling	Dysbiosis with increased *Streptococcus* and *Campylobacter*; *C. gracilis* independently associated with aortic aneurysm formation/progression	Taxon-level signal linked to aneurysm phenotype; potential risk stratification within TAK
Desbois et al. 2021 [22]	TAK, GCA, healthy donors	Peripheral blood microbiome profiling	Distinct “vasculitis” blood microbiome signature with enrichment of selected Proteobacteria and Actinobacteria vs. controls	Exploratory systemic microbial signal; hypothesis-generating for LVV biomarker development
Guleria et al. 2015 [125]	TAK vs. healthy controls	Serum NMR metabolomics	Metabolomic profile discriminates TAK from controls; partial association with activity-related metabolites	Proof-of-principle metabolomic discrimination; potential adjunct monitoring tool
Kumar et al. 2020 [126]	TAK (active vs. inactive)	Targeted serum NMR metabolomics	Circulatory glutamine/glucose ratio differentiates active from inactive disease	Activity-tracking candidate biomarker; supports immune–metabolic readout
Maughan et al. 2025 [129]	Large-vessel vasculitis spectrum (TAK/GCA)	Serum proteomics	Shared signatures of innate immune activation, complement involvement, and stromal/ECM remodeling across LVV	Prioritizes shared pathways for mechanistic follow-up; links immune activation to remodeling
Matsumoto et al. 2025 [130]	Systemic vasculitides including LVV	Trans-omics	Convergent alterations in matrix proteins, inflammatory mediators, and metabolic enzymes; MMP-12 identified as signature molecule	Integrative remodeling module; supports pathway prioritization/target discovery

TAK—Takayasu arteritis; GCA—giant cell arteritis; LVV—large-vessel vasculitis; NMR—nuclear magnetic resonance; ECM—extracellular matrix; MMP—matrix metalloproteinase; 16S rRNA—16S ribosomal RNA.

**Table 3:** Microbiota-targeted products: definitions, mechanisms, and translational considerations in aortic disease (evidence with emphasis on AAA). Evidence level reflects the availability of human AAA endpoint trials.

Feature/Type	Prebiotics	Probiotics	Synbiotics	Postbiotics	Live Biotherapeutic Products (LBPs)
Definition	Non-digestible food components selectively utilized by host microorganisms to confer a health benefit.	Live microorganisms administered to support a health-associated microbiome.	Combination of a prebiotic and a probiotic designed to enhance survival and activity of administered strains.	Preparations of non-viable microorganisms and/or their components (including microbe-derived metabolites).	Live organisms administered to prevent, treat, or diagnose disease (pharmaceutical-grade, regulated development).
Examples	Dietary fiber; inulin.	*Lactobacillus* spp.; *Bifidobacterium* spp.; *Saccharomyces boulardii*.	Probiotic strains combined with fiber/oligosaccharides.	SCFA-containing formulations; inactivated microbial preparations/components.	Candidate taxa/consortia under development (e.g., *Akkermansia muciniphila* as a candidate; strain-/formulation-dependent).
Mechanistic rationale (microbiome-focused)	Supports SCFA-producing commensals; increases SCFA biosynthesis; may improve gut barrier function.	Competitive exclusion of potential pathobionts; reinforcement of barrier integrity; promotion of anti-inflammatory microbial functions/metabolites (strain-specific).	Synergistic support of administered strains and resident commensals; potential amplification of SCFA-related functions; reductions in systemic inflammatory biomarkers reported in non-aortic settings.	Delivers bioactive microbial components/metabolites without viable organisms; may modulate Treg responses and reduce NET-related inflammation in preclinical settings.	Direct ecosystem “replacement”/augmentation with selected strains; theoretically targeted reshaping of microbial functions, but requires controlled delivery and dosing.
Potential relevance to AAA/aortic disease	Preclinical AAA data support mechanistic plausibility (barrier function, SCFA-related pathways); human aortic endpoints not established.	Indirect plausibility via inflammation/barrier modulation and cardiometabolic risk factor pathways; AAA-specific efficacy remains unproven.	Indirect plausibility via improved inflammatory and cardiometabolic profiles; AAA-specific endpoints largely untested.	Preclinical AAA work supports metabolite-centered approaches (e.g., propionate/butyrate-related mechanisms); human aortic endpoints not established.	High translational potential in principle, but currently limited aortic disease-specific evidence and substantial development challenges.
AAA-specific evidence (clinical)	Preclinical only; no human AAA endpoint trials.	No AAA trials; indirect evidence only.	No AAA trials; indirect evidence only.	Preclinical only; no human AAA endpoint trials.	Early-stage; no robust AAA trials.
Key limitations/caveats	Heterogeneous “dose” and fiber type; adherence and baseline diet confounding.	Strain-, dose-, and formulation-specific effects; caution in severe barrier dysfunction/immunosuppression depending on product.	Requires careful matching of components; variable formulations; uncertain transferability to aortic outcomes.	Product definition varies across studies; standardization and dosing of metabolites/components needed.	Manufacturing, formulation/encapsulation, dosing, stability, and regulatory constraints; strain selection and safety monitoring required.
Key sources	Bishehsari et al. 2018 [147]; Guo et al. 2025 [106]; Shremo Msdi et al. 2025 [176]	Chandrasekaran et al. 2024 [167]; Tian et al. 2022 [45]	Smolinska et al. 2025 [168]; Askari et al. 2021 [169]; Zhang et al. 2025 [170]; Dolatkhah et al. 2025 [171]	Hijová 2024 [172]; Yang et al. 2022 [173]; Tian et al. 2022 [45]	Min et al. 2025 [174]; Brittain et al. 2025 [175]

AAA—abdominal aortic aneurysm; SCFA—short-chain fatty acid; Treg—regulatory T cells; LBPs—live biotherapeutic products.

## Data Availability

No new data were created or analyzed in this study. Data sharing is not applicable to this article.

## Abbreviations

The following abbreviations are used in this manuscript:

16S rRNA	16S ribosomal RNA
AAA	abdominal aortic aneurysm
AD	aortic dissection
ALA	α-linolenic acid
Ang II	angiotensin II
CD4	cluster of differentiation 4 (CD4^+^ T lymphocytes)
CD8	cluster of differentiation 8 (CD8^+^ T lymphocytes)
CITED2	CBP/p300-interacting transactivator with ED-rich tail 2 (gene)
CRP	C-reactive protein
CVD	cardiovascular disease
DHA	docosahexaenoic acid
DNA	deoxyribonucleic acid
ECM	extracellular matrix
EPA	eicosapentaenoic acid
EPAS1	endothelial PAS domain-containing protein 1 (gene; HIF-2α)
FDR	false discovery rate
FXR	farnesoid X receptor
GCA	giant cell arteritis
GPR41	G protein-coupled receptor 41 (FFAR3)
GPR43	G protein-coupled receptor 43 (FFAR2)
HLA	human leukocyte antigen
IFN-γ	interferon gamma
IL-1β	interleukin 1 beta
IL-6	interleukin 6
IL-12	interleukin 12
IL-17	interleukin 17
IL-17A	interleukin 17A
IL-18	interleukin 18
LBPs	live biotherapeutic products
LDL	low-density lipoprotein
LPS	lipopolysaccharide
LVV	large-vessel vasculitis
MMP	matrix metalloproteinase(s)
MMP-2	matrix metalloproteinase-2
MMP-9	matrix metalloproteinase-9
MMP-12	matrix metalloproteinase-12
MR	Mendelian randomization
NET	neutrophil extracellular trap(s)
NK	natural killer (cells)
NMR	nuclear magnetic resonance
Nrf2	nuclear factor erythroid 2-related factor 2
Olfr78	olfactory receptor 78
PD-1	programmed cell death protein 1
RNA	ribonucleic acid
ROS	reactive oxygen species
SARS-CoV-2	severe acute respiratory syndrome coronavirus 2
SCFA	short-chain fatty acid(s)
TAA	thoracic aortic aneurysm
TAK	Takayasu arteritis
TGR5	Takeda G protein-coupled receptor 5
Th1	T helper 1 (cell subset)
Th17	T helper 17 (cell subset)
Th17.1	Th17.1 T cell subset
TMAO	trimethylamine-N-oxide
TNF-α	tumor necrosis factor alpha
Treg	regulatory T cell(s)
UDCA	ursodeoxycholic acid
VSMC	vascular smooth muscle cell(s)
